# Equity in health services use and intensity of use in Canada

**DOI:** 10.1186/1472-6963-7-41

**Published:** 2007-03-11

**Authors:** Yukiko Asada, George Kephart

**Affiliations:** 1 Department of Community Health and Epidemiology, Dalhousie University, Centre for Clinical Research, 5790 University Avenue, Halifax, Nova Scotia, B3H 1V7, Canada

## Abstract

**Background:**

The Canadian health care system has striven to remove financial or other barriers to access to medically necessary health care services since the establishment of the Canada Health Act 20 years ago. Evidence has been conflicting as to what extent the Canadian health care system has met this goal of equitable access. The objective of this study was to examine whether and where socioeconomic inequities in health care utilization occur in Canada.

**Methods:**

We used a nationally representative cross-sectional survey, the 2000/01 Canadian Community Health Survey, which provides a large sample size (about 110,000) and permits more comprehensive adjustment for need indicators than previous studies. We separately examined general practitioner, specialist, and hospital services using two-part hurdle models: use versus non-use by logistic regression, and the intensity of use among users by zero-truncated negative binomial regression.

**Results:**

We found that lower income was associated with less contact with general practitioners, but among those who had contact, lower income and education were associated with greater intensity of use of general practitioners. Both lower income and education were associated with less contact with specialists, but there was no statistically significant relationship between these socioeconomic variables and intensity of specialist use among the users. Neither income nor education was statistically significantly associated with use or intensity of use of hospitals.

**Conclusion:**

Our study unveiled possible socioeconomic inequities in the use of health care services in Canada.

## Background

Universal health care systems aim to provide health services based on need. Among many forms of the provision of universal health care systems around the world, the Canadian system is one of the most ambitious with public financing for all physician and hospital services deemed medically necessary with no payment at the point of service. Since the establishment of the Canada Health Act in 1984, which set criteria of public administration, comprehensiveness, universality, portability, and accessibility, supporters of the Canadian health care system have been a strong advocate for equal access for equal need. They have striven to remove financial or other barriers to access to physician and hospital services.

To what extent has the Canadian health care system met this goal of equitable access? In a comprehensive review of the Canadian literature on equity in health care in 1993, Birch and Abelson concluded that studies from 1970s and 1980s examining need and health care use showed an association between greater need and greater use but called for further research carefully examining the role of socioeconomic variables in a relationship between health care need and health care utilization [1]. Looking at recent studies, the diagnosis is promising on the surface. As one would expect, given the strong association between socioeconomic status and health, most recent Canadian studies have shown that people in lower socioeconomic status use more health care services than their counterparts. In a series of ecological studies using administrative data and the census in Winnipeg, Manitoba, for example, Roos and his colleagues found that individuals from poorer neighbourhood had higher utilization rates of physician and hospital services than those from richer neighbourhood after adjusting for age and sex [2-4]. At the individual level, using the Nova Scotia Nutrition Survey linked to administrative data Veugelers and Yip found that controlling for age and sex people with lower income were more likely to use general practitioner and hospital services than people with higher income, but there was no statistically significant relationship between income and specialist services [5]. Separate studies using the same data set showed that lower income and education were associated with higher use of physician services after adjusting for age, sex, and region [6] and higher use of physician and hospital services after adjusting for age, sex, death, and neighbourhood income and education [7].

Studies adjusting only for age and sex, however, cannot tell whether greater use of health care services by people with lower socioeconomic status is reasonable given their need for health care. Only after controlling for additional need indicators, such as health status, can we assess equal access for equal need. Studies with such adjustment have shown mixed results. Katz, Hofer, and Manning from the 1990 Ontario Health Survey found that lower income was associated with greater use of physician services [8]. However, Finkelstein, linking the Ontario portion of the 1995 National Population Health Survey (NPHS) to administrative data, and Dunlop, Coyte, and McIsaac in the 1994 NPHS found no statistically significant relationship between income and the use of general practitioner services [9,10]. In addition, Finkelstein and Dunlop and her colleagues found no relationship between income and the use of specialist services, although Dunlop and her colleagues found that women with less education used less specialist services [9,10].

Differences in results between studies may reflect a variety of factors including the data, statistical modeling strategies, and the need indicators used. Previous studies have varied considerably in the need indicators used beyond age and sex. For example, Katz and his colleagues adjusted for self-assessed health status alone [8], while Dunlop and her colleagues included additional indicators such as self-reported chronic disease and chronic disease risk factors [9]. Adjusting for differences in need by including a more comprehensive set of need indicators and allowing the effects of need indicators on health care utilization to vary by age seems logical, but this has not been done. For example, few studies [9,11] have adjusted for the presence of specific chronic health conditions. The type of chronic condition associated with ill health is likely to be an important need indicator since the capacity to benefit and the volume and type of services that may be effective varies by condition. Previous studies have also not allowed the effects of need indicators to vary by age.

Differences in study results may also reflect differences in modeling health services use (contact) versus volume of use (intensity). There are good reasons to expect that the processes determining use versus intensity of use may be different. Individual patients primarily make a decision of use or non-use, but health care providers play a major role in determining the volume of future contacts. Also, need indicators associated with having contact with the health system may be different from factors associated with the volume of contacts. To our knowledge, only a handful studies looking at health care utilization in Canada explicitly incorporated a two-stage process in analysis. They present mixed results. Some studies found no relationship between socioeconomic factors and health services use (contact) and volume of use after controlling for health care need. For example, using the 1985 General Social Survey, Birch, Eyles, and Newbold found that income and education had no effect on use or intensity of use of general practitioner services, except healthy people with lower education had greater intensity of use than healthy people with higher education [12]. Using the same data, Newbold, Eyles, and Birch found that income had no association with the intensity of hospital use and education had no effect on either use or intensity of hospital use [13]. Finkelstein in the Ontario study found that income and education had no effect on the use or intensity of use of specialist services, except education was associated with less contact with specialist services [10]. Examining use or non-use of hospital services using the 1994 NPHS, Wilkins and Park reported no relationship with education but women in the 15–39 and 40–64 age groups with inadequate income used more hospital services than those women with adequate income [11].

Other studies found some relationship between socioeconomic factors and health services use (contact) or volume of use after need adjustment. For example, in a recent Organization for Economic Cooperation and Development (OECD) study van Doorslaer and his colleagues found that lower income was associated with less contact with general practitioners and specialists and less intensity of general practitioner and specialist services use [14,15]. Dunlop and her colleagues, who examined use versus non-use and high use versus less than high use, found that lower income was associated with less contact with specialists and lower education was associated with less contact with general practitioners [9]. Newbold, Eyles, and Birch found that lower income was associated with less hospital admission [13], while van Doorslaer and his colleagues found that lower income was associated with greater hospital admission and longer hospital stays [14,15].

Building on the previous research, this study investigated the unsolved yet crucial question of whether and where socioeconomic differences in the use of health care services occur in the Canadian health care system after adjusting for need for health care. We examined all services covered under the Canada Health Act: physician (both general practitioner and specialist) and hospital services. The primary contribution of this study is the use of improved data and methodology. We used the 2000/01 Canadian Community Health Survey (CCHS), which provides a large sample size (about 110,000) and a broader set of need indicators that have been used in previous studies. In addition to adding a wide range of need indicators, we allowed the effects of chronic conditions to vary by age. As analytical methods, we employed two-part ''hurdle'' models to examine use versus intensity of use.

## Methods

### Data

Data came from the 2000/01 Canadian Community Health Survey (CCHS) [16]. This cross-sectional survey collects information on health determinants, health status, and health care utilization by personal or telephone interview. It uses a multi-stage stratified cluster sample design and collects information from one or two persons aged 12 years or older in each selected household in all provinces and territories. Excluded from the sampling frame are people living on Indian Reserves and in institutions, Canadian Forces Bases, and some remote areas, who are estimated to account for 2% of the Canadian population aged 12 years or older. The target total sample size for the 2000/01 CCHS is 133,300, and the response rate is 84.7%. We excluded subjects under aged 20 and residing in the northern territories as modeling health services use in these groups requires additional considerations. After excluding observations missing the dependent variables (see below), the sample size for our analysis was 110,923 for the contact with general practitioner services, 89,151 for the intensity of general practitioner services use, 111,087 for the contact with specialist services, 31,819 for the intensity of specialist services use, 111,104 for the contact with hospital services, and 11,291 for the intensity of hospital use.

### Variables

The dependent variables were health care utilization measured by self-reported numbers of visits or stays in the year prior to the survey. The use of general practitioners was measured by the number of visits to family doctor or general practitioner; the use of specialists by the number of visits to specialists such as surgeons, allergists, orthopaedists, gynaecologists, or psychiatrists; and the use of hospitals by the number of overnight stays as a patient in a hospital, nursing home, or convalescent home. For each type of utilization, two dependent variables were constructed. The first was a binary variable indicating use versus non-use, and the second was a variable indicating the number of visits or the number of overnight stays for those who had at least one contact.

The main independent variables of interest were socioeconomic status measured by household income and education. Household income was in five categories adjusted for the household size by Statistics Canada. Education was measured by the highest education level of the respondent: less than secondary school graduation, secondary school graduation and no or some post-secondary education, and post-secondary degree or diploma.

Following the health care utilization model proposed by Andersen and Newman [17], we adjusted health care utilization for need factors, factors that predispose individuals to health care use, factors that enable individuals to use health care, health behaviour, and health services system factors as listed in Table 1. Of special note in Table 1 is the use of province of residence as a proxy for health services system factors. The CCHS does not provide information on resources or organization of health services system, thus, we used this proxy. Inserting province as dummy variables into analysis is equivalent to using a fix-effect model, where provincial dummies capture variations in health care utilizations between provinces [18].

**Table 1:** Description of independent variables

*Predisposing factors*	
Age	Age group: 3 = 20–24 years, 4 = 25–29 years, 5 = 30–34 years, 6 = 35–39 years, 7 = 40–44 years, 8 = 45–49 years, 9 = 50–54 years, 10 = 55–59 years, 11 = 60–64 years, 12 = 65–69 years, 13 = 70–74 years, 14 = 75–79 years, 15 = 80+ years
Sex	0 = female, 1 = male
Minority	0 = white or not stated, 1 = visible minority
Education	Highest level of education: 1 = less than secondary education, 2 = secondary graduate and no or some post-secondary education, 3 = post-secondary graduate, 4 = currently in school, 99 = missing

*Enabling factors*	

Home ownership	Whether a member of household owns the dwelling: 0 = no, 1 = yes, 99 = missing
Household income	Household income adjusted for the number of people living in the household: 1 = lowest income, 2 = lower middle income, 3 = middle income, 4 = upper middle income, 5 = highest income, 99 = missing
Self-help group participation	Attendance at self-help group: 0 = no or not stated, 1 = yes
Sense of belonging to community	Strong sense of belonging to local community: 0 = no, 1 = yes, 99 = missing

*Need factors*	

Health Utilities Index	Health states, continuous: 0 = death, 1 = perfect health
Self-perceived health	1 = excellent, 2 = very good, 3 = good, 4 = fair or poor, missing = 99
Stress	Amount of stress in most days: 0 = not at all or not very stressed, 1 = a bit stressed, 2 = quite a bit or extremely stressed, 99 = missing
Depression	Periods during which the respondent felt sad or depressed or lost interest in everyday things within the past 12 months, categorical: 0 = no or mild depressive symptoms, 4 = maximum, 99 = missing
Activities of daily living (ADL)	0 = no activity limitation or not stated, 1 = sometimes has activity limitation, 2 = often has activity limitation
Instrumental activities of daily living (IADL)	Needs help for preparing for meals, shopping for necessities, housework, heavy household chores, personal care, or moving about inside the house: 0 = no, 1 = yes, 99 = missing
Birth	Has given birth in the past 5 years: 0 = no, 1 = yes
Injury	Injured in the past 12 months: 0 = no, 1 = yes
Number of chronic conditions	Number of chronic conditions: 0 = 0, 1 = 1, 2 = 2, 3 = 3, 4 = 4+, 99 = missing
Food allergies	0 = no or not stated, 1 = yes
Other allergies	Allergies other than food allergies: 0 = no or not stated, 1 = yes
Asthma	0 = no or not stated, 1 = yes
Arthritis	Arthritis or rheumatism: 0 = no or not stated, 1 = yes
High blood pressure	0 = no or not stated, 1 = yes
Migraine	0 = no or not stated, 1 = yes
Urinary incontinence	0 = no or not stated, 1 = yes
Diabetes	0 = no or not stated, 1 = yes
Chronic bronchitis	Chronic bronchitis, emphysema, or chronic obstructive pulmonary disease: 0 = no or not stated, 1 = yes
Heart disease	Heart disease or angina: 0 = no or not stated, 1 = yes
Cancer	0 = no or not stated, 1 = yes
Stomach or intestinal ulcers	0 = no or not stated, 1 = yes
Bowel disorders	Bowel disorder or Crohn's disease or colitis: 0 = no or not stated, 1 = yes
Eye problem	Cataracts or glaucoma: 0 = no or not stated, 1 = yes
Thyroid condition	0 = no or not stated, 1 = yes
Epilepsy	0 = no or not stated, 1 = yes
Chronic fatigue syndrome	Chronic fatigue syndrome, multiple chemical sensitivities, or fibromyalogia: 0 = no or not stated, 1 = yes
Stroke	Suffers from the effects of a stroke: 0 = no or not stated, 1 = yes

*Health behaviour*	

Overweight	0 = underweight or acceptable weight, 1 = overweight, 99 = missing
Smoking	0 = never smoked, 1 = former smoker, 2 = current smoker, 99 = missing
Drinking	0 = not regular drinker, 1 = regular drinker, 99 = missing
Physical activity	1 = moderate or inactive, 2 = active, 99 = missing
Fruit and vegetable consumption	Total daily fruit and vegetable consumption: 1 = less than 5 servings per day, 2 = 5+ servings per day, 99 = missing
Alternative health care	Consulted alternative health care provider (e.g., acupuncturist, homeopath, or massage therapist) for physical, emotional or mental health for the past 12 months: 0 = no or not stated, 1 = yes

*Health services system*	

Province of residence	10 = Newfoundland, 11 = Prince Edward Island, 12 = Nova Scotia, 13 = New Brunswick, 24 = Quebec, 35=Ontario, 46 = Manitoba, 47 = Saskatchewan, 48 = Alberta, 59 = British Columbia

Most of the variables included missing values. We imputed missing values for the Health Utilities Index (about 1.4% of the sample) using regression imputation with variables whose spearman correlation coefficient with the Health Utilities Index was greater than 0.10. When possible, we assigned missing values to logical existing categories. For example, missing values for self-reported specific chronic conditions were coded as not having the condition. For variables with multiple categories, we created an additional missing category. For example, we assigned those who did not have income information to a missing category (about 10% of the sample) and those who were currently in school and those who did not have education information to separate categories (about 1.48% and 1.09% of the sample). This strategy avoids exclusion of data but may bias parameter estimates. Accordingly, we also conducted sensitivity analyses with missing data excluded and found that this did not affect study conclusions.

### Analysis

We conducted separate analysis for general practitioner, specialist, and hospital use. For each, we estimated two-part ("double hurdle") models. The two-part model separates contact, use or non-use, from intensity, volume or frequency of use among the users [19,20]. The two-part model applied to different types of health services use is conceptually appealing because it explicitly acknowledges different decision-makers and need processes at different stages of health care utilization. This approach has the potential to identify where and why socioeconomic differences in health services use occur. In addition, it is methodologically attractive since it accounts for the high prevalence of zero (i.e., non-users) that characterizes health care utilization data. For the first part, we used a logistic regression model for use and non-use. For the second part, we used zero-truncated negative binomial regression analysis for frequency of use among the users [21]. The frequency of use variables are non-negative, non-zero count data, for which Poisson regression (or zero-truncated Poisson regression) is a candidate. However, the Poisson model assumes that the mean equals to the variance and that every count is independent from each other. The variance of health care utilization data often exceeds the mean, as one visit to a physician or one stay at a hospital may relate to the subsequent visits or stays. The zero-truncated negative binomial model relaxes the independence assumption and allows for over-dispersion.

All coefficients in the models were estimated using maximum likelihood estimation. All reported analyses were weighted to adjust for unequal probabilities of selection; however, unweighted analyses were also run for comparison. Weighting did not affect the primary findings. Because the public-use version of the CCHS does not contain the information necessary to obtain bootstrapped standard errors, reported standard errors do not fully account for the complex sample design. Our standard errors were estimated with robust methods, which accounted for unequal variance but not correlated observations. Given that we did not fully account for the design effect, we considered variables with a statistical significance at the 1% level as significant and retained interaction terms with this significance level in the model. The Wald statistic provided the statistical significance of each variable. To allow the effects of need indicators to vary by age, we tested for interactions between age and each of 15 specific chronic conditions. Also, to test for sex differences, we included interaction terms between sex and each of the socioeconomic status variables, education and income. While not reported, we also estimated stratified models to further explore sex differences. For the zero-truncated negative binomial model, we examined the overdispersion parameter α. We used Stata 9 for all analyses [22].

## Results

Table 2 presents health care utilization in the12 months prior to the survey among Canadians based on the 2000/01 CCHS. About 80% of the population had at least one visit to general practitioners, about 30% of the population had at least one visit to specialists, and about 9% of the population had at least one overnight stay at a hospital. Table 3 reports results of the two-part model estimations for general practitioner, specialist, and hospital services. In all models socioeconomic effects are need-adjusted.

**Table 2:** Health care utilization in 12 months

	General practitioners	Specialists	Hospital overnight stay
Mean visit (median)	3.37 (2)	0.84 (0)	0.59 (0)
% no use	20.75	70.31	91.33
% at least 1 use	79.25	29.69	8.67

All figures are weighted

**Table 3:** Two-part models of general practitioner, specialist, and hospital services

	General practitioners		Specialists		Hospitals	
			
	Logistic	Zero-truncated negative binomial	Logistic	Zero-truncated negative binomial	Logistic	Zero-truncated negative binomial
						
Variable	OR	IRR	OR	IRR	OR	IRR
Age	***	***	***	***	***	***
20–24	1.00	1.00	1.00	1.00	1.00	1.00
25–29	1.18	1.01	0.97	1.31 **	1.06	0.92
30–34	0.89	0.89 **	1.04	1.14	0.89	1.04
35–39	0.73 **	0.83 ***	0.83 *	1.06	0.72 **	1.17
40–44	0.72 ***	0.76 ***	0.73 ***	1.01	0.62 ***	0.96
45–49	0.68 ***	0.75 ***	0.76 **	0.86	0.58 ***	1.26
50–54	0.80 *	0.76 ***	0.82 *	0.86	0.59 ***	1.62 **
55–59	0.81 *	0.72 ***	0.77 **	0.78 **	0.68 **	1.67 **
60–64	0.79 *	0.68 ***	0.72 ***	0.85 *	0.71 **	1.45 *
65–69	0.73 *	0.56 ***	0.45 ***	0.60 ***	1.02	2.02 **
70–74	0.88	0.53 ***	0.43 ***	0.51 ***	0.92	2.33 ***
75–79	0.81	0.58 ***	0.43 ***	0.52 ***	1.07	2.29 ***
80+	0.96	0.61 ***	0.27 ***	0.46 ***	1.18	2.73 ***
Sex	0.35 ***	0.74 ***	0.44 ***	1.00	0.67 **	1.20
Sex × age^^	***	***	***	*	***	
Minority	1.26 ***	1.14 ***	0.95	0.73 ***	0.76 ***	0.90
**Education**	*	***	***			
**Post-secondary graduate**	**1.00**	**1.00**	**1.00**	**1.00**	**1.00**	**1.00**
**Secondary graduate**	**0.99**	1.04 **	0.91 ***	**0.98**	**1.01**	**0.98**
**Less than secondary education**	0.90 **	1.08 ***	0.74 ***	0.91 *	**1.05**	**0.96**
**Currently in school**	**1.10**	**0.92**	**1.03**	**1.09**	**0.74**	**1.58**
**Missing**	**1.01**	**1.07**	**0.86**	**0.95**	**1.01**	**1.18**

Home ownership		***		***	**	*
Own the dwelling	1.00	1.00	1.00	1.00	1.00	1.00
Do not own the dwelling	0.96	1.08 ***	0.99	1.15 ***	1.14 **	1.13 **
Missing	0.70	1.02	0.82	0.82	1.37	1.00
**Household income**	***	**	***			*
**Highest**	**1.00**	**1.00**	**1.00**	**1.00**	**1.00**	**1.00**
**Upper middle**	0.93 *	1.06 **	0.87 ***	**0.98**	**0.97**	**1.07**
**Middle**	0.83 ***	1.06 **	0.79 ***	**0.92**	**0.94**	1.17 *
**Lower middle**	0.79 ***	1.11 ***	0.76 ***	**0.90**	**0.93**	1.25 **
**Lowest**	0.70 ***	1.12 **	0.67 ***	**0.97**	**1.06**	1.29 *
**Missing**	0.87 **	**1.05**	0.83 **	**0.96**	**0.95**	1.21 *
Self-help group participation^	1.30 **	1.18 ***	1.36 ***	1.28 ***	1.34 ***	1.41 ***
Sense of belonging to community	**					
Yes	1.00	1.00	1.00	1.00	1.00	1.00
No	0.88 ***	0.97 *	0.96	1.01	0.94	1.04
Missing	0.64 **	0.91	0.91	0.80	1.00	0.86

Health Utilities Index	0.95	0.93 ***	0.91 **	0.91 *	1.04	0.99
Self-perceived health	***	***	***	***	***	***
Excellent	1.00	1.00	1.00	1.00	1.00	1.00
Very good	1.21 ***	1.21 ***	1.17 ***	1.19 ***	1.00	1.10
Good	1.25 ***	1.53 ***	1.38 ***	1.44 ***	1.39 ***	1.36 ***
Fair or poor	1.51 ***	2.00 ***	1.81 ***	1.87 ***	1.99 ***	1.80 ***
Missing	0.82	1.54 *	0.62	0.99	4.11 *	1.36
Stress	***	***				***
Not at all or not very stressed	1.00	1.00	1.00	1.00	1.00	1.00
A bit stressed	1.07 *	1.03	1.02	1.00	0.90 **	0.92
Quite a bit or extremely stressed	1.16 ***	1.10 ***	1.05	1.06	0.94	0.92 ***
Missing	1.31	0.99	0.93	1.34	0.76	1.45 *
Depression	***	***	***	***	***	***
0	1.00	1.00	1.00	1.00	1.00	1.00
1	0.96	1.17 **	1.10	1.05	1.19	0.93
2	1.33 ***	1.30 ***	1.31 ***	1.44 ***	1.34 ***	1.32 ***
3	1.92 ***	1.47 ***	1.56 ***	1.74 ***	1.73 ***	1.34 **
4	1.91 ***	1.78 ***	1.61 ***	2.03 ***	2.67 ***	1.98 ***
Missing	0.90	1.19 ***	1.13	1.39 ***	1.43 **	1.03
ADL	**	***	***	***	***	*
No limitation	1.00	1.00	1.00	1.00	1.00	1.00
Sometimes	0.98	1.08 ***	1.11 **	1.13 **	0.94	0.93
Often	1.19 **	1.17 ***	1.35 ***	1.20 ***	1.19 **	1.07
IADL	***	***	***	***	***	***
No	1.00	1.00	1.00	1.00	1.00	1.00
Yes	1.36 ***	1.35 ***	1.41 ***	1.40 ***	1.83 ***	1.50 **
Missing	1.76	1.09	1.42	1.35	1.52	1.22
Birth^	1.06	1.53 ***	1.24 ***	2.11 ***	5.26 ***	1.03
Injury^	2.30 ***	1.29 ***	1.43 ***	1.11 **	1.75 ***	1.09
Injury × age^^	**	***				
Number of chronic conditions	***	***	***	**	**	*
0	1.00	1.00	1.00	1.00 **	1.00	1.00
1	1.52 ***	1.32 ***	1.67 ***	1.17 **	1.41 ***	1.20 **
2	2.05 ***	1.57 ***	2.29 ***	1.20 **	1.48 ***	1.27 **
3	2.44 ***	1.73 ***	2.67 ***	1.26 ***	1.56 ***	1.33 **
4+	2.72 ***	1.83 ***	3.47 ***	1.48 ***	1.50 ***	1.31 **
Missing	1.04	1.74 ***	2.16 ***	1.20	1.41 *	1.32
Food allergies^	0.86 *	0.93 **	0.84 ***	0.95	0.95	1.00
Other allergies^	0.91 *	0.93 ***	0.86	0.97	0.96	0.92 *
Other allergies × age^^			***			
Asthma^	1.10	1.04 *	0.90 **	0.97	1.13 *	0.97
Arthritis^	1.19 ***	1.01	1.00	0.93 *	0.98	0.85 ***
High blood pressure^	1.94 ***	1.20 ***	0.85 ***	0.91	1.12 *	0.57
High blood pressure × age^^						**
Migraine^	0.87	1.08 **	0.89 **	0.94	0.99	0.84 **
Migraine × age^^	***					
Urinary incontinence^	1.07	0.93 *	1.13 *	0.92	1.14	1.13 *
Urinary incontinence × age^^						
Diabetes^	1.35 **	1.20	1.02	1.08	8.71 ***	1.10
Diabetes × age^^		**			***	
Chronic bronchitis^	1.12	0.99	0.84 **	0.95	1.19 **	1.04
Heart disease^	1.37 ***	1.13 ***	1.60 ***	0.93	1.91 ***	0.20 **
Heart disease × age^^						**
Cancer^	0.26	1.20 ***	3.53 ***	1.64	2.46	0.45
Cancer × age^^	***			**	***	***
Stomach or intestinal ulcers^	1.17	1.10 **	1.77 *	0.88 *	1.15	0.92
Stomach or intestinal ulcers × age^^			**			
Bowel disorders^	1.37 *	1.10	1.49 ***	1.05	1.34 ***	3.55 ***
Bowel disorders × age^^						***
Eye problem^	0.76 **	0.90 ***	0.84 ***	1.00	0.96	0.31 **
Eye problem × age^^						**
Thyroid condition^	1.63 ***	1.06 *	1.07	0.98	0.92	0.87
Epilepsy^	1.14	0.99	1.15	1.12	1.34	1.16
Epilepsy × age^^						***
Chronic fatigue syndrome^	0.93	1.05	0.94	0.98	0.84 *	1.18
Chronic fatigue syndrome × age^^						**
Stroke^	2.17	0.54	0.80 *	0.99	1.34 **	1.42 ***
Stroke × age^^	**	**				

Overweight		***	**	***	*	
Underweight or acceptable weight	1.00	1.00	1.00	1.00	1.00	1.00
Overweight	1.06 *	1.03 *	0.99	1.00	1.02	1.03
Missing	0.97	1.33 ***	1.22 **	1.34 ***	0.78 *	0.90
Smoking	***		***			
Never smoked	1.00	1.00	1.00	1.00	1.00	1.00
Former smoker	1.04	1.02	1.14 ***	1.01	1.09	1.05
Current smoker	0.77 ***	1.00	0.90 **	0.98	0.98	1.07
Missing	1.03	0.88	0.91	0.93	1.24	0.65
Drinking	**	***		***	***	***
Not regular drinker	1.00	1.00	1.00	1.00	1.00	1.00
Regular drinker	1.09 **	0.89 ***	1.04	0.85 ***	0.74 ***	0.97 ***
Missing	1.43 *	0.81 *	0.97	1.37	1.33	0.91
Physical activity	**	**			***	
Active	1.00	1.00	1.00	1.00	1.00	1.00
Moderate or inactive	1.08 *	1.06 ***	1.04	1.05	1.18 ***	0.99
Missing	1.51 **	1.12	1.10	1.33	1.27	1.38
Fruit and vegetable consumption	**	***	***		*	
5+ servings a day	1.00	1.00	1.00	1.00	1.00	1.00
Less than 5 servings a day	0.94 *	0.92 ***	0.88 ***	0.97	0.91 **	0.97
Missing	0.75 **	0.88 *	0.78 *	0.96	0.79	0.78
Alternative health care^	1.43 ***	1.20 ***	1.28 ***	1.11 **	0.92	1.00

Province of residence	**	**	***	**	**	**
ON	1.00	1.00	1.00	1.00	1.00	1.00
NF	1.59 ***	1.20 ***	1.04	0.92	1.29 **	1.22 *
PEI	1.05	0.93 *	0.88 *	0.97	1.48 ***	1.27 **
NS	1.00	1.03	0.91 *	0.80 ***	1.02	1.06
NB	1.23 ***	0.90 ***	1.02	0.71 ***	1.40 ***	1.34 ***
QC	0.66 ***	0.74 ***	1.50 ***	0.80 ***	1.34 ***	0.96
MB	0.84 ***	0.99	0.90 *	1.02	1.18 *	1.08
SK	1.12 *	1.06 *	0.79 ***	0.81 **	1.38 ***	1.00
AL	0.92 *	1.08 **	0.71 ***	0.87 **	1.17 **	1.05
BC	1.03	1.14 ***	0.75 ***	1.03	1.05	1.03

*α*		0.95		2.99		1.64
*n*	110923	89151	111087	31819	111104	11291
Pseudo-R square	0.12		0.11		0.15	
Log likelihood	-49891.01	-38209980	-60018.27	-11517957	-27813.50	-5128804.50
Wald chi-square~	4847.42 ***	13954.43 ***	6072.91 ***	2619.61 ***	5297.16 ***	2795.83 ***

^ The reference group is the absence of the condition or no use.

^^ Fifteen interaction terms are included. The coefficients are not reported due to the limited space. The reported statistical significance is overall.

* 0.01 p < 0.05, ** 0.001 p < 0.01, *** p < 0.001, Given that we did not fully account for the design effect, we considered variables with a statistical significance at the 1% level as significant.

Statistical significance in the row of a variable name indicates overall significance of all categories combined including missing category.

~ Wald chi-square test of the joint significance of the repressors.

### General practitioners

Education was not statistically significantly associated with the contact with general practitioners but was positively associated with the intensity of general practitioner services. Income was statistically significantly associated both with the contact with and the intensity of general practitioner services, and, strikingly, the direction of their graded associations was opposite in the logistic and zero-truncated negative binomial model. People with lower income were less likely to contact general practitioners than their counterparts, but, once they made the initial contact, they were likely to visit them more often.

Most of other variables, including chronic conditions such as high blood pressure, migraine, diabetes, heart disease, cancer, eye problem, and stroke were statistically significant predictors both of the contact with and the intensity of general practitioner services. However, the sense of belonging to community, smoking, arthritis, and thyroid condition were associated only with contact with general practitioners, while home ownership, the Health Utilities Index, having given birth in the past 5 years, overweight, food allergies, other allergies, and stomach or intestinal ulcers were only associated with the intensity of the use. The effects of migraine, diabetes, cancer, and stroke varied by age. Neither education nor income interacted with sex.

### Specialists

People with lower education and lower household income were less likely to contact specialists than their counterparts, but education and income had no statistically significant relationship with the intensity of specialist services.

Most of other variables were statistically significantly associated with either the contact or intensity of specialist services use. The sense of belonging to community, stress, physical activity, and some chronic conditions (arthritis, urinary incontinence, diabetes, thyroid condition, epilepsy, chronic fatigue syndrome, and stroke) were not associated with specialist services use or intensity of use. Having a lower Health Utilities Index, having smoked, and having 5 or more servings of fruits and vegetables a day were positively associated with the contact with specialists but not the intensity of use, while being a minority, owing the dwelling, and being a regular drinker were negatively associated with the intensity of specialist use but not with the contact with specialists. Many chronic conditions, such as food allergies, other allergies, asthma, high blood pressure, migraine, chronic bronchitis, heart disease, stomach or intestinal ulcers, bowel disorders, and eye problem, were only associated with the contact with specialist services. The effects of other allergies, cancer, and stomach or intestinal ulcers varied by age. The interaction term between sex and education was statistically significant in the model of the intensity of specialist services. In a separate analysis stratified by sex, we found that among men education was overall statistically significant (p = 0.004), but this was due to the category of "currently in school" (the incidence rate ratio: 1.88, p = 0.006).

### Hospital

Socioeconomic status had no statistically significant association with hospital use or non-use (hospital admission) and the intensity of use (hospital stay). All variables but the sense of belonging to community, Health Utilities Index, overweight, smoking, fruit and vegetable consumption, the use of alternative health care, and some chronic conditions (food allergies, other allergies, asthma, urinary incontinence, stomach or intestinal ulcers, eye problems and thyroid condition) were statistically significantly associated both or either of hospital admission or stay. Being female, non-visible minority, having given birth in the past 5 years, being moderately active or inactive, and having injury, diabetes, and chronic bronchitis were positively associated with hospital admission, while they had no association with hospital stay. On the other hand, stress, arthritis, high blood pressure, migraine, epilepsy, and chronic fatigue syndrome was a predictor of hospital stay though it was not for hospital adminission. Effects of many chronic conditions on hospital use varied by age, including high blood pressure, diabetes, heart disease, cancer, bowel disorders, epilepsy, and chronic fatigue syndrome. Effects of education and income did not vary by sex.

## Discussion

This study examined whether and where socioeconomic differences in need-adjusted use of general practitioner, specialist, and hospital services occur in Canada using two-part models. One of the attractive features of this approach is explicit recognition of differences in processes affecting use versus the intensity of use of different types of services. For example, the role of patients versus providers in determining utilization varies between use and intensity of use of different types of health services use. The type and importance of need indicators are also likely to vary between use and intensity of use of different types of health services. For example, ambulatory conditions such as allergies and arthritis will be more important drivers of general practitioner use, while heart disease and cancer will be more important drivers of need for specialist and hospital services. Moreover, some types of conditions (e.g., diabetes) may require more follow-up than others (e.g., allergies), and thus will be more strongly associated with intensity of use.

Another unique aspect of this study is the use of a broad set of need indicators, and the incorporation of interactions between particular need indicators with age. Overall, previous studies are likely to have under-adjusted for need indicators. Many previous studies have adjusted for only general measures of health status, and studies vary considerably in the types and range of need indicators used. The problem with using general measures of health status alone is that they do not recognize that different health problems may produce the same sense of health, but different need for types and intensity of health services. The capacity to benefit from care and the intensity and complexity of treatment options vary widely by type of health problem. The effects of need indicators are also likely to vary by age. For example, complications and severity of diabetes tend to increase with age, thus increasing the need for health services.

Our approach unveiled possible socioeconomic inequities at the entry to health care services. After adjustment for need, lower income was associated with less contact with general practitioners, but among those who had contact, lower income and education were associated with greater intensity of use of general practitioners. Both lower income and education were associated with less contact with specialists, but there was no statistically significant relationship between these socioeconomic variables and intensity of specialist use among the users. Neither income nor education was statistically significantly associated with use and non-use and intensity of hospital use. To obtain an overall picture of where socioeconomic differences in need-adjusted use of health services exist, it is important to explore who are the primary decision-makers regarding the contact with and the intensity of each of these three types of health services. A patient largely initiates contact with a general practitioner, while general practitioners play a major decision-making role in subsequent visits and referrals to specialists. Referrals to specialists, however, are not entirely determined by general practitioners. Effectiveness of patients' negotiation with general practitioners and geographic variation of substitution between general practitioners and specialists would also be important factors for the contact with specialists. The patient's role in the decision of hospital use, on the other hand, is likely substantially limited. Specialists play a major decision-making role in the decision to admit patients to hospital, as well as in the duration of hospital stays.

Taken together, our results show that disaggregating socioeconomic variations in health care use by type of service and contact versus intensity uncovers complex variations. Complexity in patterns of health services use by socioeconomic status is evident in the results of other studies as well. For example, Roos and Mustard found that excess use of hospital services for residents of lower income neighbourhoods in Winnipeg, Manitoba reflected higher admission for medical, not surgical reasons, and higher admissions for conditions that are avoidable or which can be managed in an ambulatory care setting [4]. Other Canadian studies have shown that residents of lower income neighbourhoods in Ontario [23] and Quebec [24] have lower rates of cardiac catherization and revasculariszation following admission for an acute myocardial infarction. Yet, premature mortality due to cardiovascular disease is higher among lower socioeconomic groups [25,26].

What we can conclude is that socioeconomic status is associated with how and when patients contact the health care system, but further study is needed to disaggregate the reasons for our findings. For example, our results for general practitioner services suggest that lower socioeconomic status may be associated with contacting the system later in the stages of disease severity or symptom severity; however, if this is the case, one might expect to see higher use of specialist or hospital services associated with lower SES. This was not the case. Future work needs to examine socioeconomic variation in the use of different types of specialist and hospital services (e.g. medical versus surgical admissions) to explicate the reasons for the effects we observe.

To further explain where socioeconomic differences in need-adjusted use of health services occur, future work needs to further explore socioeconomic differences in referral patterns, and the reasons for those differences. Dunlop and her colleagues controlled for the probability of using general practitioner services six times or more for the past year in their analysis of use and non-use of specialist services and frequent (6+) and less frequent use of specialist services [9]. This approach may help to adjust differences in specialist use for differences in the possibility of referral, but more detailed understanding of the process is needed. For example, we need to know if socioeconomic differences in referral to specialists reflect general practitioner's decisions, effectiveness of a patient's negotiation with the general practitioner to see specialist, or substitution between general practitioners and specialists reflecting geographic differences in access.

Our results confirm findings of some previous studies. Our study replicates the positive association between income and the contact with general practitioners found by van Doorslaer and his colleagues [14,15]. Our finding of the positive association between socioeconomic status and the contact with specialists also confirms findings by three other studies [9,10,14,15]. Like Finkelstein [10], we found no statistically significant relationship between socioeconomic status and the intensity of specialist use. As to the contact with hospitals, our study confirms Wilkins and Park for no relationship with education [11].

However, our other results are not consistent with previous findings [9,12,14,15]. Discrepancies between the study by van Doorslaer et al. and our study are particularly worth investigating given that their study used the same data and the same type of statistical modeling approach: a two-part "hurdle" model (logistic and zero-truncated negative binomial regression models). van Doorslaer and his colleagues found negative associations between income and hospital admission and stay while we found no such relationships. A primary difference between these studies is the extent of need adjustment. While van Doorslaer and his colleagues only used self-perceived health and activity status (i.e., impact of health problems on home, work or school, and other activities) as need indicators, we used more comprehensive adjustments for need (see Table 1).

To what extent can differences in need adjustment explain differences in the study results between van Doorslaer et al. and us? Did the study by van Doorslaer et al. under-adjust for need indicators, or did our study over-adjust? The answers ultimately depend on how need for health services should be defined, and whether it is measured in a way that is valid and reliable. To date, the choice and mix of need indicators has in large part been governed by data availability. Data availability is becoming less of concern with data such as the CCHS, and future work must establish a conceptual framework for the choice and mix of need indicators. Such a framework must be sensitive to the measurement construct of each need indicator and, at the same time, easy to interpret for full policy potential. Using the CCHS, which offers a wide selection of need indicators, we guided our selection and mix of need indicators based on the behavioural model by Andersen and Newman [17] and used a large selection of need indicators to capture the multidimensional concept of need for health care. For example, we believed that the Health Utilities Index captures general health status of the respondent in the standardized manner while self-perceived health allows the respondent's own evaluation of the health status. It also made sense to us to include a count of chronic conditions as well as binary variables indicating the presence of each chronic condition as a count of chronic conditions can be considered as a proxy for severity or the existence of comorbidities while different chronic conditions present different types and quantities of health care need. Yet our selection of need indicators will benefit from an in-depth conceptual analysis of the choice and mix of need indicators.

This study has important limitations. First, many of the need indicators employed in the analysis are, in part, determined by health care utilization [18,27]. As a consequence some bias in the effects of need indicators and the adjustment for need can be expected. For example, self-report of chronic conditions generally follows from diagnosis by a health professional. Those with less utilization are thus less likely to report conditions. Evidence suggests that even general measures of health status may be affected by health services utilization. However, models not adjusting for chronic conditions, which are most likely to be plagued by endogeneity, did not alter the primary study findings. Second, health care utilization in this study is measured by self-report, thereby subject to recall bias [28-30]. Ideally, as Finkelstein [10] and a series of Nova Scotian studies [5-7] showed, self-reported health care utilization need to be validated or replaced by administrative data. Third, this study used cross-sectional data, which are not ideal for analysis of health care utilization. In this study, we looked at the need indicators and socioeconomic status of the respondents in the survey year and estimated their use of health care services in the previous year. To estimate contemporaneous relationships between need indicators, socioeconomic status, and health care utilization, longitudinal data are the best. Fourth, the two-part model assumes that the first visit to a physician or stay at a hospital leads to the subsequent visits [19]. This assumption may be violated in our study, as people may have had multiple health problems, each of which requires separate visits to physicians. The CCHS only provides information on the number of visits to physicians, and there is no way one can know relations of multiple visits. Finally, the standard errors in our analysis did not fully account for the design effect. Replication methods such as the bootstrap or the jackknife would be preferred [31]. The public use version of the CCHS, used for this study, did not contain the necessary information to permit such procedures. However, bootstrapped standard errors are very unlikely to alter our results. The study employed a large sample size, so effects are estimated with high precision. Even if the standard errors for the socioeconomic variables of interest were 3–4 times their size, the study findings would be unaltered.

## Conclusion

This study showed that socioeconomic inequities at the entry to the health system may still exist after the inception of the Canada Health Act 20 years ago. This finding will be important in the debate on reforms to the health care system. We must continue to explore modernizing the health system to promote equitable access to health care services.

## Competing interests

The author(s) declare that they have no competing interests.

## Authors' contributions

YA and GK contributed equally to conception, design, analysis, interpretation of data, and writing the manuscript. Both authors read and approved the final manuscript.

## Pre-publication history

The pre-publication history for this paper can be accessed here:


